# The effects of rebound exercise on body mass index and balance in overweight and obese adults: a meta-analysis

**DOI:** 10.1530/EC-25-0825

**Published:** 2026-04-08

**Authors:** Lin Wang, Sihong Sui

**Affiliations:** ^1^ School of Physical Education, Daqing Normal University, Daqing, China; ^2^ Department of Sport & Leisure Studies, Hoseo University, Asan-si, South Korea

**Keywords:** rebound exercise, body mass index, balance, overweight, adult with obesity

## Abstract

**Background:**

Rebounding involves repetitive bouncing on a trampoline, a low-impact and enjoyable exercise. The purpose of this study was to explore the effects of rebound exercise on body mass index (BMI) and balance in adults who are overweight and obese.

**Method:**

This review searched Scopus, PubMed, Web of Science, and EBSCO databases for relevant articles from January 2005 to January 2025. During the procedure, the terms ‘rebound’, ‘trampoline’, ‘training’, ‘exercise’, ‘obesity’, and ‘overweight’ were applied. This review also utilised the standardised mean difference (SMD) as the index of effect size. RevMan 5.4 software was used to analyse the average difference in the selected literature data with 95% confidence interval (CI). This review has been registered on PROSPERO (No. CRD42025638300).

**Results:**

In the nine selected articles, a total of 234 participants (61 males and 173 females) aged over 18 years with a BMI ≥ 25 kg/m^2^ were included. The study outcomes revealed that rebound exercise considerably reduced the BMI (SMD = 0.48 (0.26, 0.69), *P* < 0.01, *I*^2^ = 56%) of individuals with overweight and obesity. Nevertheless, the subgroup analysis revealed different results for intervention periods under (SMD = 0.25 (−0.03, 0.53), *P* = 0.08, *I*^2^ = 21%) and over 12 weeks (SMD = 0.82 (0.48, 1.17), *P* < 0.01, *I*^2^ = 14%). Rebound exercise also notably affected balance in adults with overweight and obesity (SMD = −0.62 (−0.98, −0.26), *P* < 0.01, *I*^2^ = 0%).

**Conclusion:**

Rebound exercise exhibited the potential to reduce BMI and improve balance in overweight and obese adults. Nonetheless, only intervention plans of over 12 weeks demonstrated significantly greater effects. Consequently, rebound exercise can be employed as a programme to reduce BMI and improve balance in overweight and obese adults.

## Introduction

Obesity and overweight have become an alarming global epidemic. These health concerns are also linked to several physical and mental health complications (1). Over the past 48 years, the issue recorded a threefold prevalence increase and caused approximately 2.8 million deaths yearly (2). Given that body weight is a significant predictor of balance, adults with overweight and obesity are prone to balance deficits and falls (3, 4).

An increase in body mass index (BMI) may be associated with elevated levels of inflammatory factors, such as tumour necrosis factor alpha (TNF-*α*) and C-reactive protein (CRP), potentially leading to neuroinflammation (5, 6). Neuroinflammation can affect various brain parts and the nervous system responsible for motor control and coordination, damaging neural pathways vital to maintaining balance (7). Motor neurons are also compromised by the response, weakening muscles and diminishing coordination (8). Moreover, the impacts of neuroinflammation on the vestibular system result in dizziness, vertigo, and balance disorders (8). Neuroinflammation can also lead to difficulties for the brain to process and respond to balance-related information from the body due to diminished cognitive functions and sensory integration (9).

Aerobic exercise and dietary control are traditional non-pharmacological methods for controlling obesity, promoting weight loss (10, 11), and improving cardiovascular health (10, 11, 12). Good dietary and physical activity habits are key to preventing and managing obesity (13, 14). Rebounding involves repetitive bouncing on a trampoline, a low-impact and enjoyable exercise. Rebounding utilises the forces of gravity, acceleration, and deceleration, which can enhance cardiorespiratory fitness and elevate oxygen consumption (15). Furthermore, the activity stimulates the immune system in a specific way (15, 16). Moving up and down during rebounding enhances the transport of immune cells throughout the body through lymphatic flow. Strengthened leg muscles and bones and improved endurance are also advantages of the rebound exercise (16). Rebounding also presents a minimised risk of stress injuries as the body weight is primarily absorbed by the trampoline surface and evenly distributed throughout the body (17). Consequently, the activity is ideal for adults with overweight and obesity (17).

The influences of rebound exercise on BMI and balance are unclear. Although some studies indicated that 12 weeks of rebound exercise can effectively reduce BMI and improve balance in adults with overweight and obesity (2, 18), several others have reported inconsistent findings under different intervention conditions (19, 20). The current review predominantly explored the influences of rebound exercise on BMI and balance in overweight and obese adults. This meta-analysis also discussed secondary indicators, including fasting blood glucose (Glu), systolic blood pressure (SBP), and diastolic blood pressure (DBP).

## Methods

### Protocol and registration

The review protocol employed in this meta-analysis was registered with the International Prospective Register of Systematic Reviews on 27 January 2025 (registration number: CRD42025638300).

### Data sources and study selection

This review searched Scopus (*n* = 57), PubMed (*n* = 44), Web of Science (*n* = 47), and EBSCO (*n* = 43) databases for relevant articles from January 2005 to January 2025. The final publications were retrieved on 30 January 2025. During the search, the terms ‘rebound’, ‘trampoline’, ‘training’, ‘exercise’, ‘obesity’, and ‘overweight’ were applied. Appendix A (see section on Supplementary materials given at the end of the article) lists the search strategy and results for each database searched for this meta-analysis.

**Figure 1:**
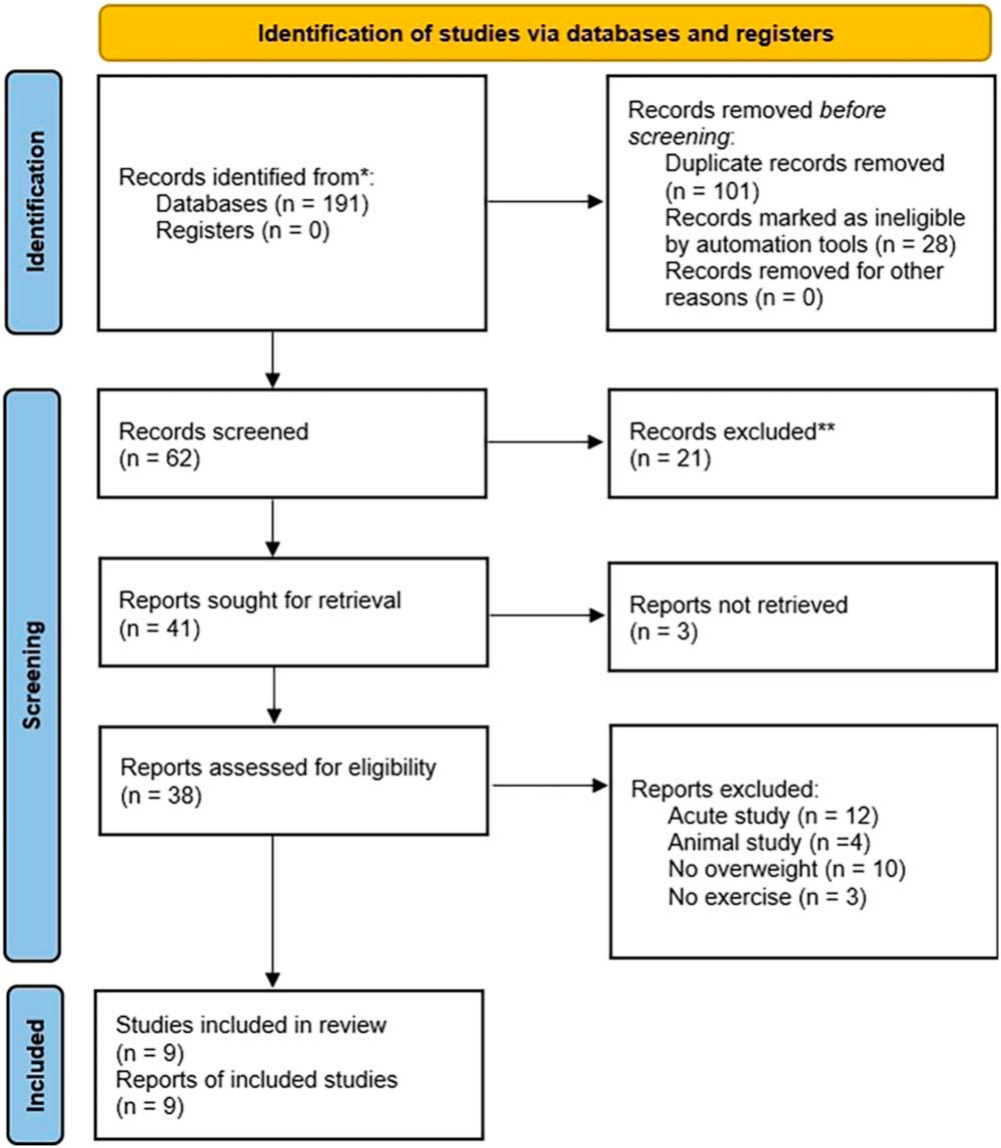
Flow diagram of the search results using the Preferred Reporting Items for Systematic Reviews and Meta-Analyses (PRISMA).

Two reviewers evaluated the selected titles and abstracts independently before screening complete articles based on the inclusion and exclusion criteria. A consensus was attained with a third investigator whenever a dispute arose between the two reviewers. Figure 1 shows the selection protocol for a systematic review based on the recommendations outlined in the Preferred Reporting Items for Systematic Reviews and Meta-Analyses (PRISMA) (21).

### Inclusion and exclusion criteria

This review included only studies on participants aged over 18 years and with a BMI ≥ 25 kg/m^2^. The articles had to report on rebound training as the intervention, overweight and obesity as the conditions, and BMI and balance as the primary outcomes. Publications that listed Glu, SBP, and DBP as secondary results and applied randomised controlled trials and single-arm studies were also included, with only pre- and post-intervention data of the rebound exercise group required for extraction. Finally, only complete articles in English with pre- and post-intervention assessment results were selected. Grey literature, including abstracts, conference proceedings, and poster presentations, was not reviewed, owing to its potential for data incompleteness and inherent methodological limitations that may compromise the reliability and robustness of the meta-analysis results. Due to insufficient randomised controlled trials and inconsistent intervention protocols for the control group, this review is a single-arm meta-analysis that excludes a control group.

### Quality assessment

In this study, the Cochrane risk-of-bias assessment tool (22) was employed to establish the methodological standard of the selected articles. The instrument was chosen as it offers several bias determinations, including random sequence generation, allocation concealment, blinding of participants, personnel, and outcome assessors, incomplete outcome data, and selective reporting (22). Each article assessed was assigned an ‘yes’, ‘no’, or ‘unclear’ score according to its quality. In this study, the Grading of Recommendations Assessment, Development, and Evaluation (GRADE) system was employed to grade the quality of evidence for the included outcome indicators, with the assessment process conducted in reference to the GRADE guidelines (23). The evidence quality was categorised into four levels: high, moderate, low, and very low (Appendix B).

### Risk-of-bias assessment

In this study, the stability of the findings was assessed through sensitivity analysis, where a publication was excluded at a time. Meanwhile, the publication bias of this review was determined via funnel plots.

### Statistical analysis

Every applicable outcome variable in the current review was input in the Review Manager (version 5.4.1, Copenhagen: The Nordic Cochrane Center, The Cochrane Collaboration, 2020). This review also utilised the standardised mean difference (SMD) as the index of effect size, considering that varying test units and equipment were reported, even though continuous outcome variables were recorded (24).

In order to evaluate the heterogeneity, *I*^2^ statistics are calculated and explained as follows: *I*^2^ value < 30% indicates that the fixed effect model should be selected for low heterogeneity (25). A range of 30–70% indicates moderate heterogeneity, while >70% indicates high heterogeneity. In both cases, the DerSimonian–Laird random effects model (REM) (26) should be selected (25). This study also attempted to further explain the heterogeneity among articles by analysing the variables age, exercise duration, frequency, intensity, and total exercise volume. In this study, the Cohen’s kappa coefficients of the two evaluators were analysed by SPSS software (version 26.0, IBM Corp., USA), where 0 indicates that the consistency is only caused by opportunity factors, 1 indicates complete consistency, and a negative value indicates that the consistency is lower than the opportunity level.

## Results

### Eligibility of studies

Of the selected articles, nine articles documented the outcomes of rebound exercise on the BMI and balance of overweight and obese adults, with six studies reporting BMI and three documenting balance. Seven reports were randomised controlled trials with highly heterogeneous control group intervention protocols and insufficient extractable quantitative data for key outcomes, and two were single-arm study designs. Thus, only pre- and post-intervention data of the rebound exercise group were extractable for all included studies. In nine of the selected articles (Table 1), 61 males and 173 females were involved. The duration of intervention of selected articles ranged from 4 to 12 weeks. The reports must also record baseline data and final data post-intervention. The included studies also obtained ethical approval from their respective  institutions. Moreover, the two reviewers reported a Cohen’s kappa coefficient of 0.92 (*P* < 0.01).

**Table 1:** Characteristics of included studies.

Study	Age (y)	Gender	BMI (kg/m^2^)	Duration	Frequency	Rebound protocol	Index
Clement *et al.* (22)	29.0 ± 12.0	8 M/15 F	28.0 ± 6.5	8 W	4x/week	Trampoline exercise, moderate intensity, maximum height, 100 bounces	BMI, SBP, DBP
Cugusi *et al. *(29)	38.1 ± 10.5	18 F	27.6 ± 2.1	12 W	3x/week	Trampoline exercise, moderate to vigorous intensity, 40–90% HRR, 55–60 min	BMI, Glu, SBP, DBP
Maharaj & Nuhu (28)	40.5 ± 6.5	22 M/23 F	26.1 ± 4.8	9 W	3x/week	Trampoline exercise, 40–60% HR_max_, 10–15 cm height, 900–2,700 bounces	BMI, Glu, SBP, DBP
Maharaj & Nuhu (27)	36.5 ± 10.6	23 F	31.0 ± 3.2	12 W	3x/week	Trampoline exercise, 40–65% HRR, 10–15 cm height, 1,250 bounces	BMI, SBP, DBP
Nuhu & Maharaj (18)	39.7 ± 2.9	15 M/15 F	26.3 ± 1.8	12 W	3x/week	Trampoline exercise, 40–60% HRR, 10–15 cm height, 1800–2,700 bounces	BMI, Glu
Ojukwu *et al.* (2)	20.2 ± 3.0	20 F	31.9 ± 3.3	6 W	3x/week	Trampoline exercise, moderate intensity, 30–45 min	Balance
Oliveira *et al. *(20)	69.0 ± 5.0	23 F	27.0 ± 3.0	12 W	2x/week	Trampoline exercise, ankle weights of 1–2 kg, 60 min	Balance
Posch *et al. *(31)	69.6 ± 5.3	20 F	27.1 ± 3.2	12 W	2x/week	Trampoline exercise, exercise for balance and strength, 45–60 min	Balance
Shah & Parab (30)	20.8 ± 3.1	16 M/16 F	26.4 ± 1.2	4 W	3x/week	Trampoline exercise, 40–80% HR_max_, 40 min	BMI

M, male; F, female; W, weeks; HR_max_, maximum heart rate; HRR, heart rate reserve; BMI, body mass index; SBP, systolic blood pressure; DBP, diastolic blood pressure; Glu, blood glucose.

### Quality analysis

**Figure 2:**
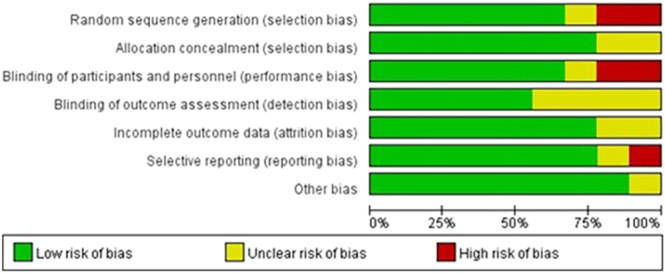
Analysis of risk of bias according to the Cochrane Collaboration guidelines.

Figure 2 illustrates the potential risk of bias and methodological quality of the nine studies reviewed. Overall, the reviewed publications had a relatively significant quality. Most of the studies also exhibited a low bias risk (73.1%), a small percentage demonstrated notable bias (7.9%), and the remaining studies were unclear (19.0%).

### Quantitative synthesis

**Figure 3:**
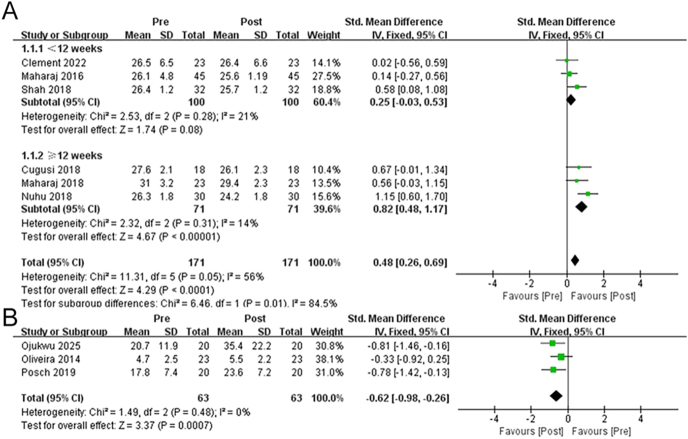
Forest plot illustrating the effects of rebound exercise on BMI (A) and balance (B).

Six (18, 19, 27, 28, 29, 30) and three (2, 20, 31) articles documented the effects of rebound exercise on BMI and balance in adults with overweight and obesity, respectively (*n* = 171 and 63). According to the results, rebounding intervention considerably reduced the BMI of (SMD = 0.48 (0.26, 0.69), *P* < 0.01) overweight and obese individuals (Fig. 3A). The reports demonstrated considerable heterogeneity (*P* < 0.05, *I*^2^ = 56%). Nevertheless, the subgroup analysis revealed different results for intervention periods under (SMD = 0.25 (−0.03, 0.53), *P* = 0.08, *I*^2^ = 21%) and over 12 weeks (SMD = 0.82 (0.48, 1.17), *P* < 0.01, *I*^2^ = 14%). Rebound exercise also notably affected balance in overweight and obese adults (SMD = −0.62 (−0.98, −0.26), *P* < 0.01) (Fig. 3B). Furthermore, the publications were non-heterogeneous (*P* = 0.48, *I*^2^ = 0%).

**Figure 4:**
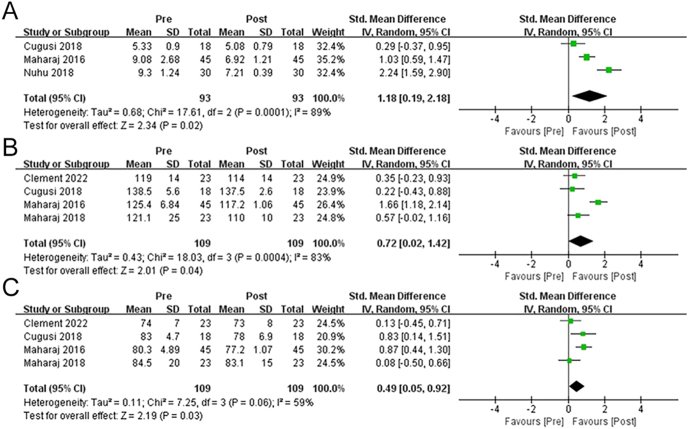
Forest plot illustrating the effects of rebound exercise on fasting blood glucose (A), systolic blood pressure (B), and diastolic blood pressure (C).

The influences of rebound exercise on Glu and blood pressure (SBP and DBP) in overweight and obese adults were evaluated in three (18, 28, 29) and four (19, 27, 28, 29) articles, respectively (*n* = 93 and 109). The activity considerably improved the Glu levels in adults with overweight and obesity (SMD = 1.18 (0.19, 2.18), *P* = 0.02)) (Fig. 4A). The studies also documented significant heterogeneity (*P* < 0.01, *I*^2^ = 89%). Similarly, notably enhanced SBP (SMD = 0.72 (0.02, 41.42), *P* = 0.04, *I*^2^ = 83%) and DBP (SMD = 0.49 (0.05, 0.92), *P* = 0.03, *I*^2^ = 59%) were observed in adults with overweight and obesity who performed rebound exercise.

### Sensitivity evaluation

No significant alterations were noted in each group following analysis type and impact size modification and exclusion of individual studies, indicating the reliability of the primary outcomes of the sensitivity analysis. Nevertheless, the poor stability of the results was recorded post-sensitivity assessment due to the notable heterogeneity of the three secondary results.

### Publication bias assessment

**Figure 5:**
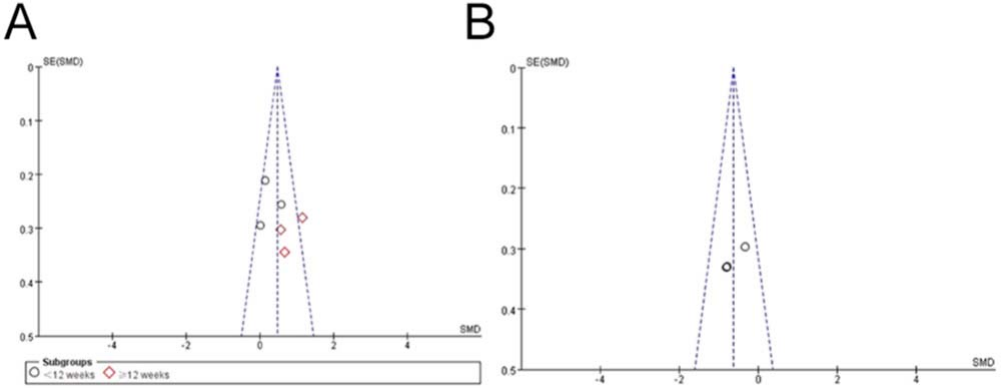
Funnel plot of publication bias for BMI (A) and balance (B) in the rebound exercise.

**Figure 6:**
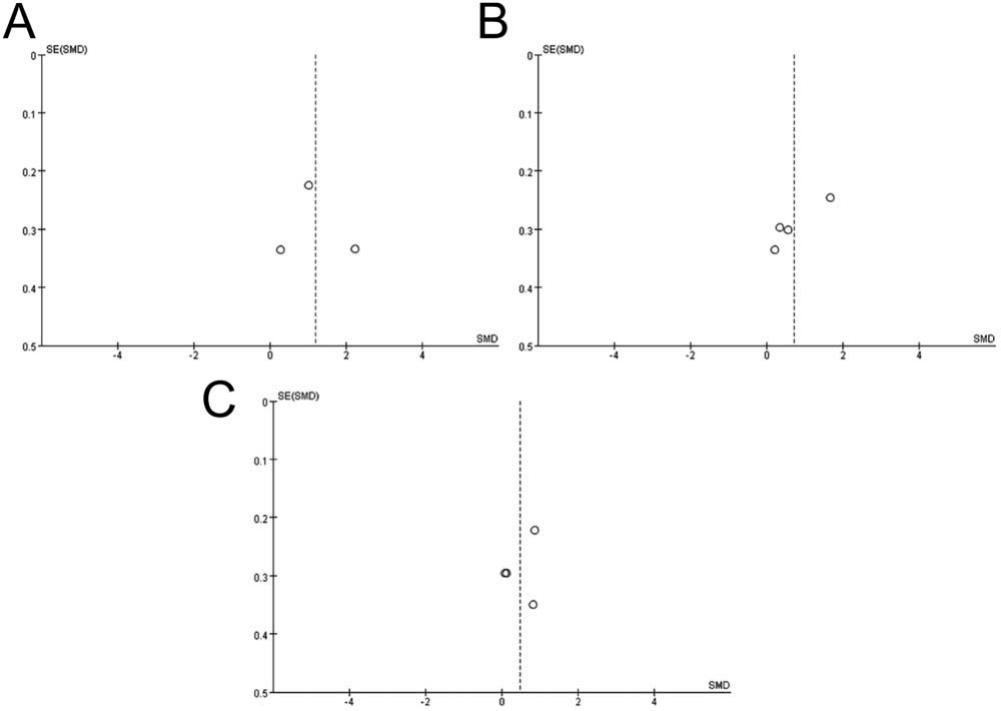
Funnel plot of publication bias for fasting blood glucose (A), systolic blood pressure (B), and diastolic blood pressure (C) in the rebound exercise.

This review analysed only nine articles, as that the sample size for rebound exercise intervention on adults with overweight and obesity was relatively small. Although the overall sample size in this study was approximately the minimum for a funnel plot analysis, the analysis was deemed appropriately reflecting a publication bias to a certain extent, with minor risks. A study reported the benefits of employing a funnel evaluation for reports involving small sample sizes (32). Figures 5 and 6 illustrate the funnel charts demonstrating the influences of rebound exercise in overweight and obese adults. Publication bias was assessed through visual interpretation of funnel plots and Egger’s test, with a significance level of *P* > 0.10.

## Discussion

This meta-analysis aimed to evaluate the advantages of rebounding on BMI and balance in overweight and obese adults. Of the nine publications reviewed, six reported BMI as the outcome measure, while three documented balance. According to the results, rebounding significantly reduced the BMI and improved the balance of the participants. Nonetheless, the BMI subgroup results varied. The participants who performed the rebound exercise for over 12 weeks had considerably reduced BMI, whereas those who performed the interventions under 12 weeks did not exhibit significant effects. The findings suggested that performing the activity for more than 12 weeks is an effective rehabilitation plan for reducing the BMI of adults with overweight and obesity.

Jumping on the trampoline, also known as rebound exercise, is becoming more and more popular as a promising treatment mode in the medical industry. Rebounding exercises leverage the bouncing effects of a trampoline to facilitate the vertical movement of the body (33). The elastic surfaces of trampolines are supported by springs and gravity, which reduce the cumulative trauma frequency from repetitive loading by minimising the jarring effects of rebounding (34). Rebounding on a trampoline offers adults with obesity a pleasurable and stimulating exercise experience by possibly masking the intensity of their physical exertion (30). Consequently, utilising a compliant trampoline for aerobic exercise presents an advantageous alternative to traditional physical activities, such as jogging, treadmill employment, interval running, home-based training, skipping, and dancing (35, 36, 37, 38, 39). In a previous meta-analysis (40), the differences in weight reduction effects of different forms of exercise were also compared. The intervention methods mainly included resistance exercise, continuous aerobic exercise, high-intensity interval exercise, and a weight-loss diet (41, 42). The results showed that as long as the energy consumption was the same, aerobic exercise and high-intensity interval training did not seem to have significant differences in weight loss, fat loss, and visceral fat reduction. However, there is still a lack of direct comparison between rebound exercise and other traditional aerobic exercises, which should be further explored in future research.

Trampolines offer considerable advantages to the human body. The contemporary apparatus facilitates rhythmic and non-rhythmic jumping exercises while mitigating joint stress (33). The activities heighten blood circulation and oxygen delivery to the cells in the body. Simultaneously, maintaining stability on the soft, unstable surface of the trampoline requires complicated sensorimotor stimulations to support balance and muscle strength (43). Consequently, utilising the equipment for exercises boosts energy levels and balance (43).

The results indicated that rebound exercise significantly improved balance in participants with overweight and obesity. Ojukwu *et al.* (2) also noted similar findings. In their report, 20 overweight women aged between 17 and 35 years were engaged in 30 min of moderate-intensity rebound exercise in the gym three times weekly for six weeks. According to the evaluation results conducted during weeks 1, 3, and 6, the balance of the individuals gradually rose over time. The study also found that rebound exercise elevated the overweight participants’ cognition considerably. Furthermore, the study noted that the participants recorded a notably increased unilateral stance period when their eyes were open but not when they were closed. With their eyes open, the participants can visually monitor their body position and make real-time adjustments, thus having better balance control (2).

Maintaining stability during rebound exercises relies heavily on visual cues, as individuals constantly adjust their position while bouncing. Consequently, the process indirectly improves balance, potentially due to enhanced visual–motor integration and proprioceptive feedback (44). The necessity to maintain balance on an unstable surface can also particularly stimulate the visual system more than the vestibular or somatosensory systems involved in postural management (45). The phenomenon could contribute to the observed predominant effects of rebounding on the visual component of postural control. Moreover, bouncing repetitively during rebound exercise can result in better dynamic balance, considering that the activity requires constantly adjusting muscle activation and joint positioning (46), stimulating the vestibular system, enhancing proprioception and neuromuscular regulation, and strengthening lower limb muscles (47).

Improved balance could be contributed partly by changes in BMI. This review found that rebound exercise, particularly after 12 weeks or more, can significantly reduce BMI levels. In a study by Cugusi *et al.* (29), 18 overweight women underwent 12 weeks of trampoline exercise. Each session lasted 55 and 60 min, which was conducted three times per week. The weight, BMI, and waist-to-hip ratio of the participants documented a significant decrease post-intervention. The results show that trampoline has a significant weight-loss-promoting effect, which supports the results of this study.

Jogging or bouncing on a trampoline is primarily vertically oriented. Nevertheless, jumping on a trampoline in one place affects metabolic output, as raising the centre of gravity commonly involves a higher metabolic cost (48). Similar observations have been reported with parallel physical activities, including treadmill walking with a graded incline and hiking-like exercises. Performing activities on a trampoline is more unstable than on the ground, resulting in higher caloric expenditure (30).

In the movement mode of rebound, most of the studies used the basic *in situ* vertical jump, and the exercise intensity was medium and high. It is divided into rhythmic jumping (28) and non-rhythmic jumping (19). In addition, a few studies (20, 31) have also supplemented the strength training in the form of squat jumping or increasing load. In view of the heterogeneity introduced by inconsistent intervention protocols, the REM was adopted to account for the observed heterogeneity across the included studies. From the results, no matter what form of rebound exercise, it can have a significant effect on BMI and balance ability.

Rebound exercise showed an extremely large effect on reducing fasting Glu (SMD = 1.18, *I*^2^ = 89%, *P* = 0.02), and the clinical significance of this SMD is population-specific: the three included studies comprised two studies (18, 28) of diabetic patients who were overweight/obese and one study on only overweight adults with normal baseline Glu (29). The extremely large SMD was driven by the significant Glu reduction in diabetic patients, as rebound exercise improves insulin sensitivity in this population (18), while the effect was mild in populations with normal Glu, which aligns with clinical metabolic principles. The extremely high heterogeneity was mainly caused by fundamental differences in study populations. Given only three included studies and high heterogeneity, this Glu outcome is an exploratory finding with limited clinical generalisation.

## Limitations

This meta-analysis has several limitations, including the small number of included articles, nine in total, and insufficient total participant numbers, 234 for BMI and balance, 93 for Glu, and 109 for blood pressure, which is a key factor leading to the downgrading of evidence quality in the GRADE assessment. The balance function assessment methods employed in the reviewed publications were also not uniform. Therefore, to minimise the impact of evaluation methods on the results, the method of SMD was adopted to compare the difference in effect sizes between the two groups. Second, high heterogeneity was detected in the secondary outcomes, including Glu (*I*^2^ = 89%, *P* < 0.01) and SBP (*I*^2^ = 83%), which substantially undermines the reliability of these findings and constitutes a major limitation of the present study. An attempt was made to explore the sources of heterogeneity through subgroup analysis or meta-regression based on age, duration, frequency, intensity, and total exercise volume. Unfortunately, no significant factors were identified to explain the heterogeneity in SBP and DBP. For Glu, only three studies were included. Although it may be affected by diabetes (Appendix C), the number of studies included is limited, and no further reliable statistical analysis could be carried out. Therefore, these secondary outcomes were considered as exploratory indicators, and their findings should be interpreted with caution. The intervention protocols reported were also inconsistent. The studies employed individual jumping protocols and combined rebound exercise procedures with other exercises on the trampoline. Due to insufficient randomised controlled trials and inconsistent intervention protocols for the control group, this review is a single-arm meta-analysis that excludes the control group.

The mechanisms of how rebound exercise reduces BMI and improves balance remain uncertain, requiring further research. Future studies might also consider conducting sufficient randomised controlled trials and including additional control groups for further data analysis. As rebound exercise may produce varying effects on individuals of different ages and diseases, future research can focus on comparing the influences of the activity on various demographics.

## Conclusion

Rebound exercise exhibited the potential to reduce BMI and improve balance in overweight and obese. Nonetheless, only intervention plans of over 12 weeks demonstrated significantly greater effects. Consequently, rebound exercise can be employed as a programme to improve BMI and balance among adults with overweight and obesity. Interpretations of these findings require caution, given the high heterogeneity and result instability in the sensitivity analysis, with secondary outcomes being exploratory only.

## Supplementary materials



## Declaration of interest

The authors declare that there is no conflict of interest that could be perceived as prejudicing the impartiality of the work reported.

## Funding

This work did not receive any specific grant from any funding agency in the public, commercial, or not-for-profit sector.

## Author contribution statement

LW wrote the main manuscript text. LW and SS prepared Figs 1, 2, 3, 4, 5, 6 and Table 1. LW and SS drafted and reviewed the manuscript.

## Data availability

The datasets used and/or analysed during the current study are available from the corresponding author on reasonable request.

## References

[1] Afshin A, Forouzanfar MH, Reitsma MB, et al. & GBD 2015 Obesity Collaborators. Health Effects of Overweight and Obesity in 195 Countries over 25 Years. N Engl J Med 2017 377 13–27. (10.1056/NEJMoa1614362) 28604169 PMC5477817

[2] Ojukwu CP, Nnyaba IS, Ede SS, et al. The effect of rebound exercise on cognition and balance of females with overweight and obesity. Libyan J Med 2025 20 2438513. (10.1080/19932820.2024.2438513) 39643930 PMC11626867

[3] Lee JJ, Hong DW, Lee SA, et al. Relationship between obesity and balance in the community-dwelling elderly population: a cross-sectional analysis. Am J Phys Med Rehabil 2020 99 65–70. (10.1097/PHM.0000000000001292) 31464747

[4] Del Porto H, Pechak C, Smith D, et al. Biomechanical effects of obesity on balance. Int J Exerc Sci 2012 5 301–320. (10.70252/ZFZP6856)

[5] Yang Y, Shields GS, Wu Q, et al. The association between obesity and lower working memory is mediated by inflammation: findings from a nationally representative dataset of U.S. adults. Brain Behav Immun 2020 84 173–179. (10.1016/j.bbi.2019.11.022) 31785398

[6] Lentoor AG. Obesity and neurocognitive performance of memory, attention, and executive function. NeuroSci 2022 3 376–386. (10.3390/neurosci3030027) 39483430 PMC11523749

[7] Bersano A, Engele J & Schäfer MKE. Neuroinflammation and brain disease. BMC Neurol 2023 23 227. (10.1186/s12883-023-03252-0) 37308838 PMC10258999

[8] Schmidt MI, Duncan BB, Sharrett AR, et al. Markers of inflammation and prediction of diabetes mellitus in adults (atherosclerosis risk in communities study): a cohort study. Lancet 1999 353 1649–1652. (10.1016/s0140-6736(99)01046-6) 10335783

[9] Cassiano LMG, Oliveira MS, Pioline J, et al. Neuroinflammation regulates the balance between hippocampal neuron death and neurogenesis in an ex vivo model of thiamine deficiency. J Neuroinflammation 2022 19 272. (10.1186/s12974-022-02624-6) 36376954 PMC9664832

[10] Al-Mhanna SB, Rocha-Rodriguesc S, Mohamed M, et al. Effects of combined aerobic exercise and diet on cardiometabolic health in patients with obesity and type 2 diabetes: a systematic review and meta-analysis. BMC Sport Sci Med Rehabil 2023 15 165. (10.1186/s13102-023-00766-5) PMC10696788 38049873

[11] Batrakoulis A, Jamurtas AZ, Metsios GS, et al. Comparative efficacy of 5 exercise types on cardiometabolic health in overweight and obese adults: a systematic review and network meta-analysis of 81 randomized controlled trials. Circ Cardiovasc Qual Outcomes 2022 15 e008243. (10.1161/CIRCOUTCOMES.121.008243) 35477256

[12] Al-Mhanna SB, Franklin BA, Jakicic JM, et al. Impact of resistance training on cardiometabolic health-related indices in patients with type 2 diabetes and overweight/obesity: a systematic review and meta-analysis of randomised controlled trials. Br J Sports Med 2025 59 733–746. (10.1136/bjsports-2024-108947) 39773835

[13] Netz Y. Is there a preferred mode of exercise for cognition enhancement in older Age? A narrative review. Front Med 2019 6 57. (10.3389/fmed.2019.00057) PMC6450219 30984760

[14] Langoni CDS, Resende TDL, Barcellos AB, et al. Effect of exercise on cognition, conditioning, muscle endurance, and balance in older adults with mild cognitive impairment: a randomized controlled trial. J Geriatr Phys Ther 2019 42 E15–E22. (10.1519/JPT.0000000000000191) 29738405

[15] Kaka B & Maharaj SS. Effect of rebound exercises and circuit training on complications associated with type 2 diabetes: protocol for a randomized controlled trial. JMIR Res Protoc 2018 7 e124. (10.2196/resprot.8827) 29735476 PMC5962829

[16] Rathi MA, Joshi R, Munot P, et al. Rebound exercises in rehabilitation: a scoping review. Cureus 2024 16 e63711. (10.7759/cureus.63711) 39099935 PMC11296216

[17] Bhattacharya A, McCutcheon EP, Shvartz E, et al. Body acceleration distribution and O_2_ uptake in humans during running and jumping. J Appl Physiol 1980 49 881–887. (10.1152/jappl.1980.49.5.881) 7429911

[18] Nuhu JM & Maharaj SS. Influence of a mini-trampoline rebound exercise program on insulin resistance, lipid profile and central obesity in individuals with type 2 diabetes. J Sports Med Phys Fitness 2018 58 503–509. (10.23736/S0022-4707.17.07120-1) 28249384

[19] Clement TJ, Alexander KV & Draper N. The effect of high intensity, short duration trampolining on human physiology across an 8- week intervention. J Sport Exerc Sci 2022 6 147–152. (10.36905/jses.2022.03.01)

[20] de Oliveira MR, da Silva RA, Dascal JB, et al. Effect of different types of exercise on postural balance in elderly women: a randomized controlled trial. Arch Gerontol Geriatr 2014 59 506–514. (10.1016/j.archger.2014.08.009) 25239512

[21] Hutton B, Salanti G, Caldwell DM, et al. The PRISMA extension statement for reporting of systematic reviews incorporating network meta-analyses of health care interventions: checklist and explanations. Ann Intern Med 2015 162 777–784. (10.7326/M14-2385) 26030634

[22] Higgins JPT, Thomas J, Chandler J, et al. In Cochrane Handbook for Systematic Reviews of Interventions. Eds JPT Higgins, J Thomas, J Chandler, Cumpston M, Li T, Page M, Welch V. Wiley, 2019. (10.1002/9781119536604) PMC10284251 31643080

[23] Guyatt G, Oxman AD, Akl EA, et al. GRADE guidelines: 1. Introduction-GRADE evidence profiles and summary of findings tables. J Clin Epidemiol 2011 64 383–394. (10.1016/j.jclinepi.2010.04.026) 21195583

[24] Li S, Shaharudin S, Cirer-Sastre R, et al Effects of high-intensity interval exercise on cardiac troponin elevation when comparing with moderate-intensity continuous exercise: a systematic review and meta-analysis. PeerJ 2023 11 e14508. (10.7717/peerj.14508) 36647447 PMC9840388

[25] Higgins JPT, Thompson SG, Deeks JJ, et al. Measuring inconsistency in meta-analyses. BMJ 2003 327 557–560. (10.1136/bmj.327.7414.557) 12958120 PMC192859

[26] DerSimonian R & Laird N. Meta-analysis in clinical trials. Control Clin Trials 1986 7 177–188. (10.1016/0197-2456(86)90046-2) 3802833

[27] Maharaj SS & Nuhu JM. Mini-trampoline rebound exercises: a “self-care” initiative for glycated hemoglobin, body mass index and emotional distress for mildly Obese females with non-insulin dependent type 2 diabetes. Diabetes Metab Syndr 2019 13 1569–1573. (10.1016/j.dsx.2018.11.006) 31336523

[28] Maharaj SS & Nuhu JM. Rebound exercise: a beneficial adjuvant for sedentary non-insulin-dependent type 2 diabetic individuals in a rural environment. Aust J Rural Health 2016 24 123–129. (10.1111/ajr.12223) 26255814

[29] Cugusi L, Manca A, Serpe R, et al. Effects of a mini-trampoline rebounding exercise program on functional parameters, body composition and quality of life in overweight women. J Sports Med Phys Fitness 2018 58 287–294. (10.23736/S0022-4707.16.06588-9) 27441918

[30] Shah M & Parab SA. Effect of rebound exercises in overweight individuals on BMI, waist-hip ratio and lung functions : randomized control trial. Int J Sci Res Sci Technol 2018 4 1837–1843. (https://ijsrst.com/IJSRST1845501)

[31] Posch M, Schranz A, Lener M, et al. Effectiveness of a mini-trampoline training program on balance and functional mobility, gait performance, strength, fear of falling and bone mineral density in older women with osteopenia. Clin Interv Aging 2019 14 2281–2293. (10.2147/CIA.S230008) 31908438 PMC6929928

[32] Lu Y, Wang W, Ding X, et al. Association between the promoter region of serotonin transporter polymorphisms and recurrent aphthous stomatitis: a meta-analysis. Arch Oral Biol 2020 109 104555. (10.1016/j.archoralbio.2019.104555) 31550570

[33] Okemuo AJ, Gallagher D & Dairo YM. Effects of rebound exercises on balance and mobility of people with neurological disorders: a systematic review. PLoS One 2023 18 e0292312. (10.1371/journal.pone.0292312) 37797042 PMC10553300

[34] Arabatzi F. Adaptations in movement performance after plyometric training on mini-trampoline in children. J Sports Med Phys Fitness 2017 58 66–72. (10.23736/S0022-4707.16.06759-1) 27813394

[35] James M, Todd C, Scott S, et al. Teenage recommendations to improve physical activity for their age group: a qualitative study. BMC Public Health 2018 18 372. (10.1186/s12889-018-5274-3) 29558987 PMC5859389

[36] Al-Mhanna SB, Batrakoulis A, Wan Ghazali WS, et al. Effects of combined aerobic and resistance training on glycemic control, blood pressure, inflammation, cardiorespiratory fitness and quality of life in patients with type 2 diabetes and overweight/obesity: a systematic review and meta-analysis. PeerJ 2024 12 e17525. (10.7717/peerj.17525) 38887616 PMC11182026

[37] Al-Mhanna SB, Batrakoulis A, Mohamed M, et al. Home-based circuit training improves blood lipid profile, liver function, musculoskeletal fitness, and health-related quality of life in overweight/obese older adult patients with knee osteoarthritis and type 2 diabetes: a randomized controlled trial duri. BMC Sport Sci Med Rehabil 2024 16 125. (10.1186/s13102-024-00915-4) PMC11145895 38831437

[38] Batrakoulis A, Jamurtas AZ, Georgakouli K, et al. High intensity, circuit-type integrated neuromuscular training alters energy balance and reduces body mass and fat in Obese women: a 10-month training-detraining randomized controlled trial. PLoS One 2018 13 e0202390. (10.1371/journal.pone.0202390) 30138475 PMC6107179

[39] Batrakoulis A, Jamurtas AZ, Tsimeas P, et al. Hybrid-type, multicomponent interval training upregulates musculoskeletal fitness of adults with overweight and obesity in a volume-dependent manner: a 1-year dose-response randomised controlled trial. Eur J Sport Sci 2023 23 432–443. (10.1080/17461391.2021.2025434) 34974824

[40] Bellicha A, van Baak MA, Battista F, et al. Effect of exercise training on weight loss, body composition changes, and weight maintenance in adults with overweight or obesity: an overview of 12 systematic reviews and 149 studies. Obes Rev 2021 22 e13256. (10.1111/obr.13256) 33955140 PMC8365736

[41] Khalafi M, Habibi Maleki A, Symonds ME, et al. The effects of intermittent fasting on body composition and cardiometabolic health in adults with prediabetes or type 2 diabetes: a systematic review and meta-analysis. Diabetes Obes Metab 2024 26 3830–3841. (10.1111/dom.15730) 38956175

[42] Khalafi M, Maleki AH, Ehsanifar M, et al. Longer-term effects of intermittent fasting on body composition and cardiometabolic health in adults with overweight and obesity: a systematic review and meta-analysis. Obes Rev 2025 26 e13855. (10.1111/obr.13855) 39501676

[43] Hahn J, Shin S & Lee W. The effect of modified trampoline training on balance, gait, and falls efficacy of stroke patients. J Phys Ther Sci 2015 27 3351–3354. (10.1589/jpts.27.3351) 26696696 PMC4681903

[44] Abd-Elmonem AM & Elhady HSA. Effect of rebound exercises on balance in children with spastic diplegia. Int J Ther Rehabil 2018 25 467–474. (10.12968/ijtr.2018.25.9.467)

[45] Pšeničnik Sluga S & Kozinc Z. Sensorimotor and proprioceptive exercise programs to improve balance in older adults: a systematic review with meta-analysis. Eur J Transl Myol 2024 34 12010. (10.4081/ejtm.2024.12010) 38213185 PMC11017176

[46] Heyland DK, Johnson AP, Reynolds SC, et al. Procalcitonin for reduced antibiotic exposure in the critical care setting: a systematic review and an economic evaluation. Crit Care Med 2011 39 1792–1799. (10.1097/CCM.0b013e31821201a5) 21358400

[47] Mergner T, Hlavacka F & Schweigart G. Interaction of vestibular and proprioceptive inputs. J Vestib Res 1993 3 41–57. 8275243

[48] Alexander K, Clement T & Draper N. Developing a mathematical model to predict energy expenditure while bouncing on a trampoline. Eur J Sport Sci 2021 21 141–148. (10.1080/17461391.2020.1728390) 32036776

