# Supplementary material for: The effects of rebound exercise on body mass index and balance in overweight and obese adults: a meta-analysis
Source: Endocr Connect. 2026 Apr 8;15(4):e250825. doi: 10.1530/EC-25-0825 (PMC13087868; doi:10.1530/EC-25-0825)
Supplement: Supplementary file 1 [file supplementary_materials.pdf]

Appendix A

Search strategy

Databases	Search strategy	Result
Scopus	#1: Title-Abs-Key (Rebound or Trampoline)	31,481
	#2: Title-Abs-Key (Exercise or Training)	1,417,431
	#3: Title-Abs-Key (Obese or Obesity or	512,331
	Overweight)	57
	#4: #1 and #2 and #3	
	Limiters - Published Date: 20050101-20250130	
Pubmed	#1: [Title/Abstract] Rebound or Trampoline	29,751
	#2: [Title/Abstract] Exercise or Training	998,257
	#3: [Title/Abstract] Obese or Obesity or	486,721
	Overweight	44
	#4: #1 and #2 and #3	
	Filters: Publication date from 2005/01/01 to	
	2025/01/30	
Web of	#1: TOPIC: (Rebound or Trampoline)	24,048
Science	#2: TOPIC: (Exercise or Training)	1,199,921
	#3: TOPIC: (Obese or Obesity or Overweight)	475,805
	#4: #1 and #2 and #3	47
	Refined by: PUBLICATION YEARS:	
	(20250130-20050101)	

	Indexes=SCI-EXPANDED, SSCI,<br>CCR-EXPANDED,	
EBSCO	#1: Abstract: (Rebound or Trampoline)	26,747
	#2: Abstract: (Exercise or Training)	1,061,425
	#3: Abstract: (Obese or Obesity or Overweight)	424,134
	#4: #1 and #2 and #3	43
	Year: 20050101-20250130	

Appendix B Grade evidence profile

Certainty assessment							№ of patients	Effect		Certainty	Importance
№ of studies	Study design	Risk of bias	Inconsistency	Indirectness	Imprecision	Other considerations	Theory-based interventions	Relative (95% CI)	Absolute (95% CI)		
Body mass index											
6	randomised trials	serious[a]	not serious	not serious	not serious[c]	none	171	-	SMD **0.48 higher** (0.26 higher to 0.69 higher)	⊕⊕⊕◯ Moderate	
Balance											
3	randomised trials	serious[a]	not serious	not serious	not serious[c]	none	63	-	SMD **0.62 lower** (0.98 lower to 0.26 lower)	⊕⊕⊕◯ Moderate	
Fasting blood glucose											
3	randomised trials	serious[a]	serious[b]	not serious	not serious[c]	none	93	-	SMD **1.18 higher** (0.19 higher to 2.18 higher)	⊕⊕◯◯ Low	
Systolic blood pressure											
4	randomised trials	serious[a]	serious[b]	not serious	serious[c]	none	109	-	SMD **0.72 higher** (0.02 higher to 1.42 higher)	⊕◯◯◯ Very low	
Diastolic blood pressure											
4	randomised trials	serious[a]	serious[b]	not serious	serious[c]	none	109	-	SMD **0.49higher** (0.05 higher to 0.92 higher)	⊕◯◯◯ Very low	

CI: confidence interval; **SMD:** standardised mean difference

a. Downgrade one level for most of the study have unclear or high risk of bias
b. Downgrade one level for the inconsistency due to considerable heterogeneity ($I^2 > 50\%$)
c. Downgrade one level for the imprecision due to the total sample size does not meet the rules of thumb (at least 400 participants

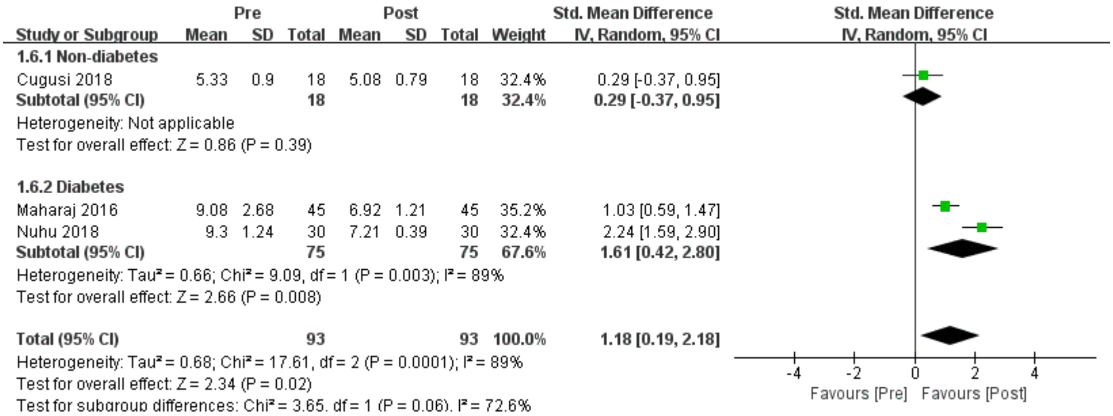

Study or Subgroup	Pre Mean	Pre SD	Pre Total	Post Mean	Post SD	Post Total	Weight	Std. Mean Difference IV, Random, 95% CI
**1.6.1 Non-diabetes**								
Cugusi 2018	5.33	0.9	18	5.08	0.79	18	32.4%	0.29 [-0.37, 0.95]
**Subtotal (95% CI)**			**18**			**18**	**32.4%**	**0.29 [-0.37, 0.95]**
Heterogeneity: Not applicable								
Test for overall effect: Z = 0.86 (P = 0.39)								
**1.6.2 Diabetes**								
Maharaj 2016	9.08	2.68	45	6.92	1.21	45	35.2%	1.03 [0.59, 1.47]
Nuhu 2018	9.3	1.24	30	7.21	0.39	30	32.4%	2.24 [1.59, 2.90]
**Subtotal (95% CI)**			**75**			**75**	**67.6%**	**1.61 [0.42, 2.80]**
Heterogeneity: Tau² = 0.66; Chi² = 9.09, df = 1 (P = 0.003); I² = 89%								
Test for overall effect: Z = 2.66 (P = 0.008)								
**Total (95% CI)**			**93**			**93**	**100.0%**	**1.18 [0.19, 2.18]**
Heterogeneity: Tau² = 0.68; Chi² = 17.61, df = 2 (P = 0.0001); I² = 89%								
Test for overall effect: Z = 2.34 (P = 0.02)								
Test for subgroup differences: Chi² = 3.65, df = 1 (P = 0.06), I² = 72.6%								

Appendix C. Forest plot illustrated the effects of rebound exercise on fasting blood glucose.
